# Control of Gut Inflammation by Modulation of Purinergic Signaling

**DOI:** 10.3389/fimmu.2020.01882

**Published:** 2020-09-25

**Authors:** Marta Vuerich, Samiran Mukherjee, Simon C. Robson, Maria Serena Longhi

**Affiliations:** ^1^Department of Anesthesia, Critical Care & Pain Medicine, Beth Israel Deaconess Medical Center, Harvard Medical School, Boston, MA, United States; ^2^Division of Gastroenterology, Department of Medicine, Beth Israel Deaconess Medical Center, Harvard Medical School, Boston, MA, United States

**Keywords:** adenosine receptor, P2 receptor, ectonucleotidase, crohn's disease, ulcerative colitis

## Abstract

Inflammatory bowel disease (IBD) is a serious inflammatory condition of the gastrointestinal tract. Crohn's disease (CD) and ulcerative colitis (UC) are two of the most common IBD manifestations and are both associated with unfettered inflammation, often refractory to conventional immunosuppressive treatment. In both conditions, imbalance between effector and regulatory cell immune responses has been documented and is thought to contribute to disease pathogenesis. Purinergic signaling is a known modulator of systemic and local inflammation and growing evidences point to extracellular ATP/adenosine imbalance as a key determinant factor in IBD-associated immune dysregulation. *In vitro* and pre-clinical studies suggest a role for both ATP (P2) and adenosine (P1) receptors in dictating onset and severity of the disease. Moreover, our experimental data indicate ENTPD1/CD39 and CD73 ectoenzymes as pivotal modulators of intestinal inflammation, with clear translational importance. Here we will provide an updated overview of the current knowledge on the role of the purinergic signaling in modulating immune responses in IBD. We will also review and discuss the most promising findings supporting the use of purinergic-based therapies to correct immune dysregulation in CD and UC.

## Introduction

Healthy tissues contain negligible levels of extracellular nucleotides and nucleosides; whereas inflammatory sites are characterized by accumulation of extracellular ATP and adenosine.

Release of nucleotides in the extracellular environment triggers P2 receptors activation on target cells. The P2 receptor family includes seven P2X and eight P2Y members, classified based on their desensitization time and affinity for the ligand. P2 receptors are virtually present on all immune cells, are mainly activated by extracellular ATP and have been generally described as mediators of inflammatory processes (1).

Once released in the extracellular environment, nucleotides can be rapidly hydrolyzed into nucleosides by specific ectonucleotidases. Ectonucleotidases are expressed on the surface of different immune cell types and belong to several enzymatic families, which have been functionally and structurally characterized (2, 3). The prototype member of the NTPDase family is ENTPD1/CD39, a rate-limiting ectoenzyme that hydrolyzes ATP into AMP, which is then further degraded into adenosine by the ecto-5′-nucleotidase/CD73 (2).

**Figure 1:**
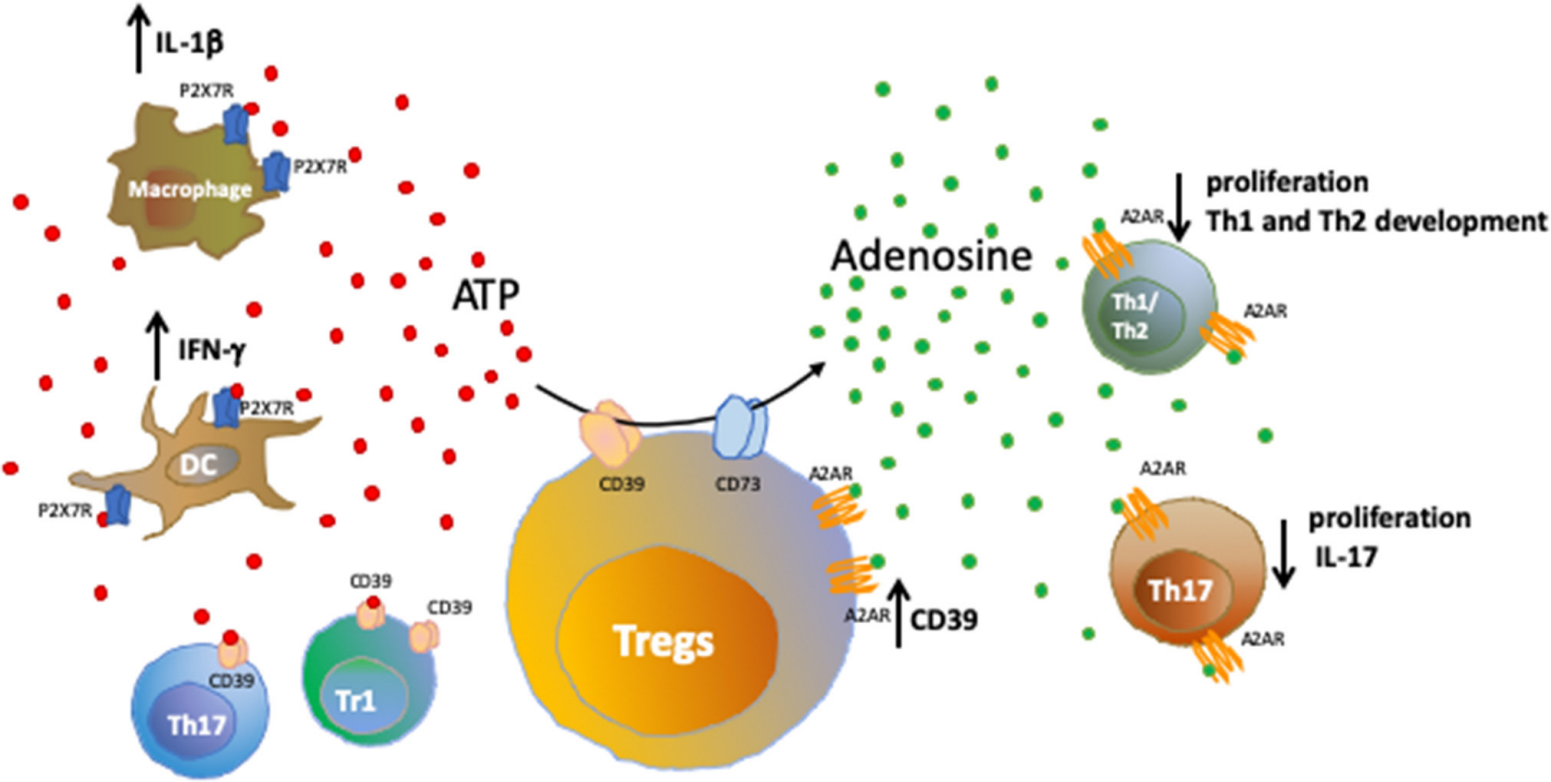
Purinergic signaling in immune cells. Healthy tissues contain negligible levels of extracellular nucleotides; whereas inflammatory sites are characterized by accumulation of extracellular ATP that binds purinergic receptors on target cells, further amplifying inflammatory responses. We show here the effects resulting from the activation of ATP-P2X7R axis in macrophages and dendritic cells. Extracellular nucleotides can be converted into nucleosides by ectonucleotidases present on Tregs, T regulatory type-1 (Tr1) cells and a subset of Th17-cells. The activity of CD39 and CD73 ectoenzymes expressed by Tregs and converting pro-inflammatory ATP into anti-inflammatory adenosine is shown. Upon binding to the A2A receptor (A2AR), extracellular adenosine suppresses effector T cell responses, leading to reduced cell proliferation, limited Th1 and Th2 development and control of IL-17 production by Th17-cells. Notably, A2AR is expressed also by Tregs and its activation promotes CD39 expression.

Once generated, extracellular adenosine can activate P1 receptors (adenosine receptors) on target cells. Adenosine receptors are classified into four subtypes (A1, A2A, A2B, and A3) and consist of G-coupled, 7-transmembrane spanning receptors expressed by a wide range of immune cells. Adenosine receptors have been mainly associated with immunoregulatory functions (4). Figure 1 shows how the purinergic signaling modulates immune responses during inflammation.

In this review, we will discuss the role of the purinergic signaling, with a focus on P1 and P2 receptors and on ENTPD1/CD39 and CD73 ectoenzymes, in the context of inflammatory bowel disease (IBD). We will report the most important findings obtained from human studies and experimental models of colitis, and we will also highlight potential novel therapeutic approaches, such as administration of exogenous recombinant ectonucleotidases and targeting of specific intracellular pathways that interfere with purinergic signaling.

IBD is a chronic inflammatory condition of the gastrointestinal tract associated with altered gut microbial composition, disrupted mucosa structure and systemic biochemical alterations (5, 6). IBD most frequent manifestations include Crohn's disease (CD) and ulcerative colitis (UC), which are diagnosed based on the localization of intestinal inflammation and clinical symptoms (5, 7).

Mounting evidence has indicated Th17-cells as the main effector players involved in IBD tissue damage and several additional studies have reported the role of purinergic signaling alterations in the immunopathogenesis of the disease.

Recommended standard therapies for IBD include corticosteroids (7), immunosuppressive drugs (8, 9), amino-salicylates (10, 11), and biological agents like infliximab (12). These currently available treatments, however, are often associated with adverse effects and limited therapeutic efficacy, this emphasizing the need for novel and more effective therapies. Given the role played in the modulation of immune responses, the purinergic signaling might represent a potential therapeutic target.

## P2X Receptor Family Members - P2X7

Alterations of purinergic signaling play an important role in promoting tissue inflammation in IBD and several evidences support the involvement of P2X receptors and specifically the P2X7 receptor (P2X7R).

Systemic P2X7R inhibition by administration of the selective inhibitor A740003 or brilliant blue G, prevents the development of TNBS-induced colitis in rat models (13). Similarly, P2X7R deletion is protective in murine models of colitis (14).

**Figure 2:**
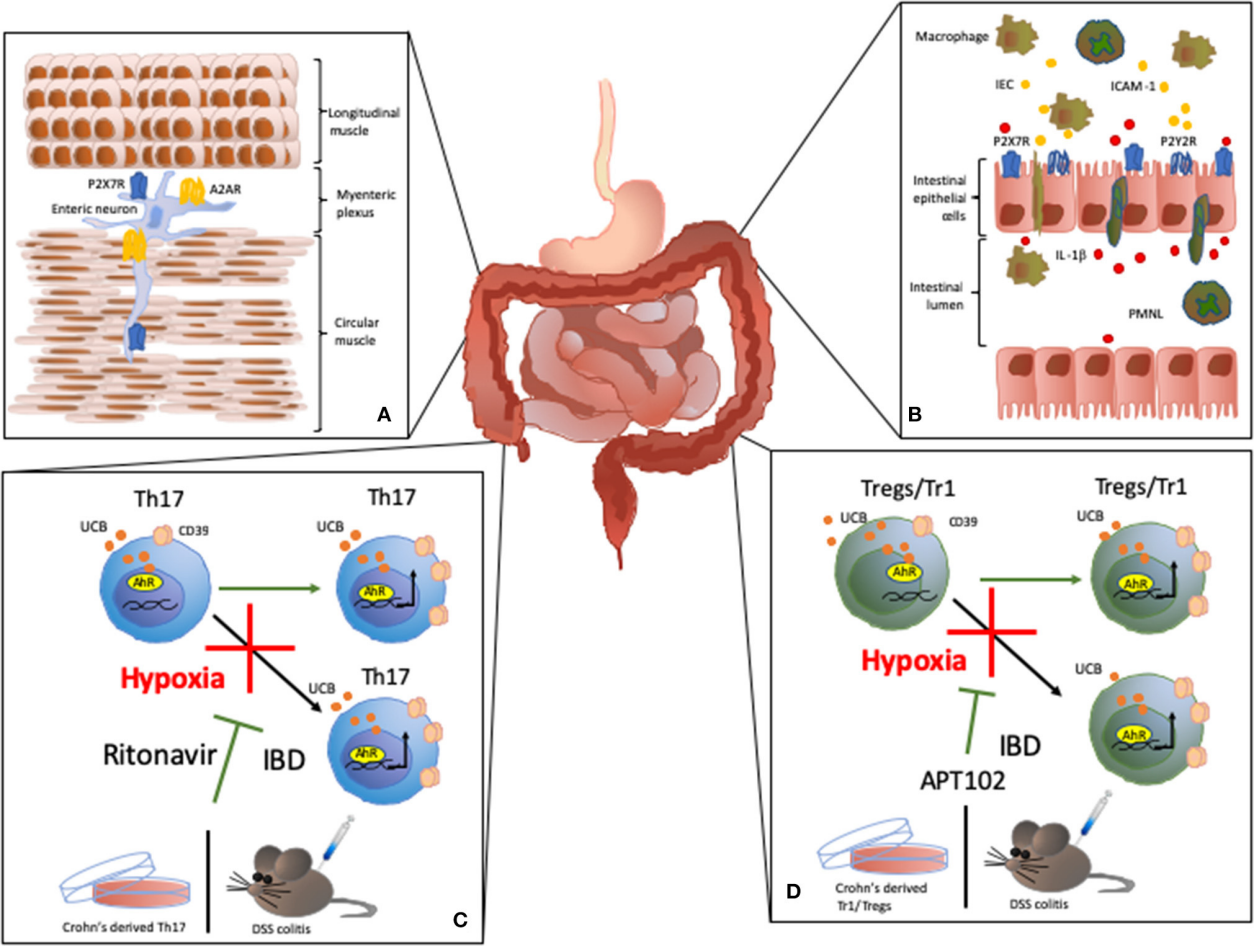
Effects of purinergic signaling modulation in IBD. **(A)** Effects on colon motility: P2X7R activity has been associated with death of enteric neurons that leads to impaired colon motility in IBD (15). A2AR activity modulates the inhibitory effect of adenosine on colonic motility during experimental colitis. **(B)** P2Y receptor (P2Y2R) expression and activation on intestinal epithelial cells (IEC) increases the expression of chemoattractive proteins, like ICAM-1, in IEC, promoting macrophage transepithelial migration and adhesion (16). In the inflamed lamina propria and gut epithelium, P2X7R activation triggers IL-1β secretion by IEC in response to polymorphonuclear leukocyte (PMNL) transmigration (17). **(C,D)** In Tr1, Treg, and Th17-cells, CD39 expression is induced upon activation of aryl hydrocarbon receptor (AhR), an intracellular receptor for pollutants, toxins and endogenous compounds, like unconjugated bilirubin (UCB). UCB boosts cell immunoregulatory properties *in vitro* and ameliorates the course of experimental colitis *in vivo* (18). However, in Crohn's disease or experimental colitis, Treg, Tr1, and Th17-cells are refractory to the regulatory effects of UCB due to a deleterious effect of hypoxia (19). Notably, we found that HIF-1α inhibition or ABC transporters blockade obtained upon administration of the antiretroviral ritonavir, limits the detrimental effects of hypoxia in Th17-cells *in vitro* in *in vivo* (19). Similarly, administration of the ADPase APT102, restores the response of Treg and Tr1-cells to the regulatory effects of UCB and ameliorates experimental colitis *in vivo* (20).

P2X7R activation has been also associated with the death of enteric neurons that lead to impaired colon motility (Figure 2A). In rat models of ulcerative colitis, P2X7R co-localizes with immunoreactive cells in the myenteric plexus (15). The decrease in neuronal density in the myenteric plexus has a positive correlation with P2X7R expression in different neuronal populations present in the area (15). P2X7R stimulation triggers pannexin-1 (Panx1) channels opening and inflammasome activation, including the Asc adaptor protein and caspase cleavage, which results in neuronal death. Accordingly, in murine models of colitis, selective inhibition of each component of the P2X7R-inflammasome axis significantly dampens the inflammation while Panx1 inhibition reduces the colonic dysfunction *in vivo* (21).

Additional evidence has indicated that in a model of 2,4-dinitrobenzenesulfonic-acid (DNBS)-induced colitis P2X7R is upregulated in the neuromuscular layer; notably, increase in electrically induced contractions was recorded in colonic preparations, obtained from colitic mice, and exposed to A804598, a selective P2X7R antagonist (22).

P2X7R expression promotes intestinal inflammation also by inducing different subsets of effector cells, including mast cells. Increased numbers of mast cells were found in the colon of CD patients and experimental murine models of colitis (23). Mast cell-deficient or P2X7R^−/−^ mast cell-reconstituted mice present lower susceptibility to inflammation as compared to the wild type counterpart. Interestingly, P2X7R-mediated activation of mast cells triggers release of pro-inflammatory cytokines, chemokines, and leukotrienes that recruit neutrophils in the inflamed area (23). Further, P2X7R activation has been linked to death and retention of regulatory T-cells (Tregs) in the mesenteric lymph nodes impairing gut immune tolerance (14).

The inflammatory process in the gut is linked to polymorphonuclear leukocyte (PMNL) transmigration into the mucosa leading to increased IL-1β production by intestinal epithelial cells (IEC). Interestingly, in CD patients, P2X7R is overexpressed in the inflamed lamina propria and gut epithelium, where P2X7R activation triggers IL-1β secretion by IEC in response to transmigration of PMNL (17) (Figure 2B).

In the intestinal mucosa, P2X7R is also present on macrophages and dendritic cells where the expression of this receptor positively correlates with IFN-γ, TNF-α, and IL-1-β levels, this leading to epithelial cells apoptosis. Concomitantly, lower concentrations of IL-10 have been also detected (24). *In vitro* experiments, conducted on human colonic mucosa strips, have revealed that blockade of Panx1 and P2X7R significantly reduce crypt damage, pro-inflammatory cytokine release, loss of tight junctions, and cell permeability (25).

It has been hypothesized that the presence of a gain of function single nucleotide polymorphism or the loss of function SNPs (His155Tyr, Arg307Gln, and Glu496Ala) affect P2X7R activity and could be associated with susceptibility to CD. However, no significant differences were noted among the subjects carrying the polymorphisms in terms of disease incidence (26). A phase II clinical trial on patients with moderate to severe CD evaluated the use of a selective P2X7R antagonist, AZD9056, as a potential therapeutic approach. Despite not having effects on the levels of inflammatory biomarkers, oral administration of AZD9056 induced an overall improvement in the disease symptoms (27). Further investigations are therefore needed to develop P2X7R antagonists that effectively interfere with the inflammatory response; in this regard, several compounds have been proposed and these include Pyroglutamide-Based P2X7R Antagonists (28).

P2X7R, however, plays also an important role in the regulation of follicular T helper cell density in Peyer's patches (29) while favoring the generation of metabolic homeostasis by sensing microbiota-derived ATP (30).

## P2Y Receptors

The G-coupled associated P2Y receptors (P2YR) are also involved in IBD immunopathogenesis. *In vitro* experiments using human nerve-gut preparations and mouse colonic sensory neurons, showed that P2YR activation triggers visceral nociceptors stimulation that can lead to the visceral pain associated with inflammation (31).

Colonic tissue isolated either from IBD patients or mice with experimental colitis displays higher expression of P2Y2 receptor (P2Y2R) when compared to healthy controls. Further investigations have revealed that increase in P2Y2R expression depends on the activity of the C/EBPβ transcription factor, which is also upregulated in IEC in murine models of colitis (32). Investigations conducted on human colon cell lines and colonic tissue from CD and UC patients, revealed that during intestinal inflammation P2YR expression is also regulated through a NF-kB p65-dependent mechanism (33). Activation of P2YR, and particularly P2Y2R, increases the expression of chemoattractive proteins, like ICAM-1, in IEC; this promoting macrophage transepithelial migration and adhesion (16) (Figure 2B).

On the other hand, experiments conducted in murine models of dextran sulfate sodium (DSS) colitis showed that administration of the P2Y2R agonist 2-thioUTP reduces the disease activity index and histological scores. These evidences suggest a role for P2Y2R in the remission phase of IBD (34).

Another study identified a specific activity of P2Y6R that was found to regulate CXCL8 expression in IEC, promoting neutrophil recruitment and inflammatory responses (35). In contrast, in murine models of DSS colitis, P2Y6R deletion has been associated with extensive intestinal inflammation, resulting from increased recruitment of Th17/Th1 lymphocytes in the gut mucosa (36).

In humans, P2Y6R is expressed in a wide range of inflammatory cells and expression levels increase in activated CD4^+^ and CD8^+^ T-cells. Because of the pro-inflammatory activity, P2Y6R has been implicated in the pathogenesis of IBD-mediated intestinal damage (37).

## ENTPD1/CD39 and CD73 Ectoenzymes

The involvement of ENTPD1/CD39 and CD73 ectoenzymes in the modulation of intestinal inflammation has been extensively studied.

In experimental mouse models of colitis, the impact of CD39 expression strictly depends on the model considered as well as on the cell populations involved in the tissue damage. In the setting of trinitro-benzene-sulfonic-acid (TNBS)-induced colitis in humanized mice, Goettel et al., demonstrated that activation of the transcription factor aryl hydrocarbon receptor (AhR)—an intracellular receptor for pollutants, toxins, and endogenous compounds—by indole-3′-carbonyl-thiazole-4-carboxylic-acid-methyl-ester, induced Tregs and this was linked to CD39 upregulation (38). In contrast, a more favorable course of TNBS-induced colitis has been observed in CD39-null mice when compared to wild type controls (39). In the context of DSS colitis, ENTPD1/CD39 and CD73 expression on activated macrophages was found to limit inflammation, either by directly hydrolyzing pro-inflammatory extracellular ATP into adenosine or by indirectly promoting Treg development (40). Further, in the same experimental model, CD39 deletion exacerbated colitis as reflected by heightened disease activity index, higher levels of pro-inflammatory markers and histological evidence of tissue injury.

Notably, in humans, the presence of SNPs associated with lower levels of CD39 expression correlate with increased susceptibility to CD (41); whereas increased CD39 levels in peripheral blood Tregs are associated with clinical and endoscopic remission (42). Recently Huang and colleagues reported multiple cellular defects in pediatric colitis and IBD, including impaired cyclic AMP-response signaling, infiltration of phosphodiesterase 4B and TNF-expressing macrophages, platelet aggregation and decrease in CD39-expressing intraepithelial T-cells (43); notably, administration of the phosphodiesterase inhibitor dipyridamole ameliorated colitis symptoms in a pilot study (43).

CD39 is known to play an important role in the immunosuppressive activity of suppressor Th17-cells (supTh17). This cell population derives from iTregs upon exposure to Th17 polarizing conditions. SupTh17-cells display high expression of CD39 and actively contribute to the production of adenosine. Compared to *bona fide* pathogenic Th17, supTh17-cells display higher expression of the enzyme adenosine deaminase and lower expression of A2A receptor (A2AR), these features making these cells refractory to the inhibitory effects of adenosine (44).

We recently found that, in human Th17-cells, CD39 expression is induced upon activation of AhR via unconjugated bilirubin (UCB). Exposure to UCB boosts Th17 immunoregulatory properties *in vitro* and ameliorates the course of experimental DSS colitis *in vivo* (18). Furthermore, Th17-cells from CD patients are refractory to the immunosuppressive effects of UCB. This lack of response is directly dependent on high levels of hypoxia-inducible-factor-1alpha (HIF-1α) that promotes ABC transporters to favor UCB exit from the cells and therefore limiting AhR activation. Interestingly, blockade of HIF-1α or ABC transporters limits the detrimental effects of hypoxia both *in vitro* and *in vivo* (19) (Figure 2C). Further, human CD39 overexpression or administration of APT102—the extracellular domain with improved ADPase activity of human nucleoside triphosphate diphosphohydrolase-3 (CD39L3), a member of the CD39 family—restores AhR-mediated regulatory effects on CD-derived Tregs *in vitro* and prevents hypoxia-related damage in experimental colitis *in vivo* (20) (Figure 2D).

There is however evidence that in IBD patients with active disease, CD73 expression in CD4^+^ T-cells is associated with a pro-inflammatory Th17-cell phenotype; based on this evidence, CD73 could be therefore used as a marker to monitor disease activity during treatment (45).

## A2AR

In IBD, the beneficial effects of adenosine generation by CD39 and CD73 ectoenzymes, have been supported by a wealth of studies. In rat models of chronic experimental colitis, administration of two different selective adenosine deaminase inhibitors significantly improved the course of disease (46). Data revealed that both compounds significantly decreased the inflammatory parameters and the beneficial effect was abrogated in the presence of pharmacological blockade of A2AR or A3 receptor (A3R), suggesting a protective role for both these receptors (46).

A study on the effects of electroacupuncture on visceral pain in a murine model of TNBS-colitis, showed that the beneficial effect of the treatment was linked to increased expression of A1R, A2AR, and A3R and to decreased expression of A2B receptor (A2BR) in colonic tissue. The antalgic effect was mediated by inhibition of release of the pro-inflammatory factors substance P (SP) and IL-1β. This salutary effect was partially abrogated in the presence of adenosine receptors antagonists (47).

A2AR activity has been associated with amelioration of spontaneous ileitis and administration of the A2AR agonist ATL-146e significantly reduced the intestinal mucosa inflammation, leukocyte infiltration in the gut and release of proinflammatory cytokines (48). A2AR plays also an important role in modulating colonic motility. In this regard, exposure of rat colonic longitudinal muscle preparations to the receptor antagonist ZM 241385 increases transmural electrical stimulation-induced contractions, whereas exposure to the receptor agonist CGS 21680, triggered a concentration-dependent reduction of contractile responses. Interestingly, these modulatory functions are further enhanced in preparations derived from animals exposed to DNBS-induced colitis (49). Similarly, in rat ileum/jejunum preparations, CGS 21680 administration prevented the TNBS-related inhibition of acetylcholine-induced contractions and A2BR antagonist PSB-1115 inhibited the contraction-decreasing effect of TNBS. The effect was even enhanced in response to combinatorial treatment with CGS 21680 and PSB-1115, both used at subthreshold concentrations (50). The beneficial role of A2AR in the context of experimental colitis has been also supported by the observation that administration of the A2AR agonist polydeoxyribonucleotide (PDRN), ameliorated the clinical symptoms and promoted tissue repair in two models developed in Sprague-Dawley rats. PDRN administration significantly reduced the circulating levels of pro-inflammatory cytokines, along with decrease in malondialdehyde and myeloperoxidase activity (51). A newly synthetized polar A2AR agonist, in which polar groups were introduced to prevent peroral absorption and subsequent systemic side effects, has been proposed as a potential treatment for IBD. Preliminary experiments conducted in rat ileum/jejunum preparations showed a significant improvement of the impaired acetylcholine-induced contractions and this beneficial effect was boosted by A2BR selective antagonists (52). Importantly, administration of this compound in a rat model of oxazolone-induced colitis limited weight loss and decreased levels of TNF-α in colonic tissue (53).

In humans, overexpression of the miRNA-16 has been reported in UC patients. A recent study identified a correlation between miRNA-16 overexpression and A2AR downregulation in the colonic mucosa of active UC patients. In the same study it was also reported that miRNA-16 inhibits the expression of the *A2AR* gene by acting at the post-transcriptional level; this effect being mediated upon engagement of the NF-κB pathway (54).

## A2BR

Despite the widely described immunoregulatory effects of adenosine, there is evidence supporting a pro-inflammatory role of A2BR in the context of intestinal inflammation.

Experiments conducted in murine models of colitis induced by DSS, TNBS, and Salmonella typhimurium showed a protective effect of A2BR knockout that was associated with lower neutrophil responses, although cell recruitment to the inflammatory site was not impacted (55). Accordingly, administration of the A2BR selective antagonist ATL-801 in DSS-treated wild type or piroxicam-treated IL-10^−/−^ mice, significantly lowered severity of colitis along with levels of pro-inflammatory cytokines (56). Engagement of A2BR has been also linked to the damage associated with intestinal ischemia reperfusion (I/R) injury and hypoxia. In this regard, administration of the A2BR antagonist PSB-1115 results in protection of the intestinal epithelial structure in a murine model of intestinal I/R and in an *in vitro* model of acute hypoxia (57). Combinatorial administration of PSB-601—another A2BR antagonist—and the A2AR agonist PSB-0777 was found to limit the TNBS-induced contractile disruption in rat ileum/jejunum preparations (52). On the other hand, deletion of A2BR in IEC has been reported having a protective role (55). As an example, an epithelial-specific A2BR deletion resulted in a milder form of experimental colitis, when compared to wild type controls. Further, *in vitro* studies have shown that the receptor activation on epithelial cells enhances a specific barrier repair response by inducing phosphorylation of vasodilator-stimulated phosphoprotein (VASP) (58).

## A3R

The A3R has been also implicated in the modulation of intestinal inflammation. In this context, there have been antithetical reports, providing evidences for either a protective or pathogenic role for this receptor during intestinal inflammation.

In a murine model of experimental colitis induced by intrarectal administration of DNBS, the beneficial effect of adenosine deaminase inhibitors was abrogated in the presence of A2AR and A3R antagonists, suggesting a protective role for both receptors (46). Further, inhibition of visceral pain by electroacupuncture in mice with TNBS-induced colitis, are accompanied by upregulation of A3R along with A2AR and A1R (47). Conversely, a study conducted in a murine model of DSS-induced colitis, revealed a pathogenic effect for the A3R. The impact of A3R deletion was evaluated on the clinical course of experimental colitis and on colon motility that was assessed upon measurement of artificial bead-expulsion, stool-frequency and FITC-dextran transit (59). Interestingly, A3R deficiency protected from DSS-induced tissue damage, limiting the CD4^+^-cell infiltration in the colon and preserving colon motility (59). The pathogenic role of A3R was also suggested by a clinical study that reported higher levels of A3R in PBMCs from patients with different autoimmune disorders, including CD, when compared to healthy subjects (60).

However, the effects of A3R expression strictly depend on the cells, in which the receptor is expressed. A study conducted on human colonic epithelial cells reported that A3R activation inhibits NF-kB signaling pathway leading to inhibition of IL-8 and IL-1β pro-inflammatory cytokines (61). In line with this observation, the use of the A3R agonist N(6)-(3-iodobenzyl)-adenosine-5-N-methyluronamide in a rat chronic model of TNBS-induced colitis showed beneficial effects on the course of the disease. Interestingly, the receptor inhibitor limited colitis-induced upregulation of other pro-inflammatory purinergic receptors like P2X1, P2X4, P2X7, P2Y2, P2Y6, as well as A2AR and A2BR (62).

Importance of the adenosinergic signaling has been further supported by the findings of Aherne et al. who showed that increased intestinal adenosine levels resulting from epithelial specific deletion of equilibrative nucleoside transporter 2 protected from inflammation in mice with experimental colitis (63).

Supplementary Table 1 summarizes the effects of purinergic receptors in IBD pathophysiology.

## Concluding Remarks

Mounting clinical evidence and research data support the involvement of purinergic signaling alterations in IBD pathogenesis, with imbalances in the ATP/adenosine ratio being regarded as underlying immunological dysregulation in this condition. As already observed in other autoimmune conditions, promising therapeutic candidates based on adenosine or ENTPD1/CD39 and CD73 ectonucleotidases have been identified.

Boosting CD39 expression either by inducing AhR-signaling or by administering exogenous ADPase, which displays ectoenzymatic activity comparable to human CD39, showed important immunoregulatory effects *in vitro* and *in vivo*, in experimental colitis models. Encouraging pre-clinical data support also the use of selective A2AR agonists, in association with specific inhibition of A2BR. Inhibition of P2X7R-mediated responses has been also associated with beneficial effects.

Taken together, the currently available evidences, implicate the use of purinergic-mediated strategies as adjunctive treatments to correct immune dysregulation in IBD patients.

## Author Contributions

MV wrote the manuscript. SM helped drafting some sections of the manuscript. SR and ML reviewed and edited the manuscript. All authors contributed to the article and approved the submitted version.

## Conflict of Interest

The authors declare that the research was conducted in the absence of any commercial or financial relationships that could be construed as a potential conflict of interest.
