# Supplementary material for: Control of Gut Inflammation by Modulation of Purinergic Signaling
Source: Front Immunol. 2020 Sep 25;11:1882. doi: 10.3389/fimmu.2020.01882 (PMC7544737; doi:10.3389/fimmu.2020.01882)
Supplement: Supplementary file 1 [file Table_1.pdf]

Supplementary Table 1. Effects of purinergic receptors in the pathophysiology of inflammatory bowel disease

Purinergic receptor	Cell population	Experimental model/clinical settings	Effect	Reference
**P2X7R**	Global deletion/inhibition	TNBS-induced colitis (rat or mouse)	Administration of selective inhibitor A740003 or brilliant blue G prevents development of experimental colitis in rat;<br>P2X7R global deletion is protective in murine models	[13,14]
	Enteric Neurons	Rat model of ulcerative colitis	Expression correlates with decreased neuronal density and is associated with enteric neuron death	[15]
		TNBS-induced colitis (rat)	Promotes intestinal inflammation	[15]
	Mast cells	TNBS-induced colitis (mouse)<br>Crohn's disease patients	Release of pro-inflammatory cytokines	[23]
	$CD4^{+}CD45RB^{low}$ regulatory T cells	TNBS-induced colitis (mouse)	Higher sensitivity to ATP-induced cell death	[14]
	Macrophages, DC	CD patients' intestinal mucosa	Release of IFN-γ, TNF-α and IL-1β	[24]
	Intestinal epithelial cells (IEC)	Colonic tissue from CD and UC patients	Expression of chemoattractive proteins (e.g. ICAM-1) and induction of IL-1β secretion	[17]
**P2Y2R**	Administration of 2-thioUTP	DSS-induced colitis (mouse)	Reduction of disease activity index, protective role	[34]
	Human colonic cell lines or human IEC	Colonic tissue from CD and UC patients	P2Y2R activation increases expression of chemoattractive proteins	[33]
**P2Y6R**	IEC	DSS-induced colitis (mouse)	Induction of CXCL8 expression with consequent neutrophil recruitment and inflammation	[35]
	Th17/Th1 cells	DSS-induced colitis (mouse)	Global deletion is associated with increased Th17/Th1 cell recruitment in the gut mucosa and increased levels of pro-inflammatory cytokines	[36]
	Activated $CD4^{+}$ and $CD8^{+}$ T-cells	Peripheral blood samples from IBD patients	P2Y6R expression is associated with activated pro-inflammatory phenotype	[37]
**A1R, A2AR, A2BR and A3R**	Murine colonic tissue	TNBS-induced colitis (mouse)	Electroacupuncture increases A1R, A2AR, A3R expression and decreases A2BR levels in colonic tissue;<br>inhibition of IL-1β and substance P (SP) release	[47]
**A2AR**	Colonic muscle	Rat colonic longitudinal muscle preparations and DNBS-induced colitis (rat)	Receptor stimulation reduces colon motility	[49]
	Global inhibition	DNBS and DSS-induced colitis in Sprague-Dawley rats	Administration of A2AR agonist polydeoxyribonucleotide (PDRN), ameliorates clinical symptoms and reduces pro-inflammatory cytokine levels	[51]
		Colonic mucosa of active CD patients	Overexpression of miRNA-16 is associated with A2AR downregulation	[54]
		Oxazolone-induced colitis (rat)	Administration of A2AR agonist PSB-0777 ameliorates clinical conditions and decreases TNF-α levels in colonic tissue	[53]
**A2BR**		Rat ileum/jejunum preparations	A2BR selective antagonists improve the impaired acetylcholine-induced contractions	[52]
	Neutrophils	DSS- or TNBS-induced colitis (mouse)	A2BR deletion is associated with lower neutrophil recruitment	[55]
		DSS- or Piroxicam-induced colitis (mouse)	Administration of A2BR selective antagonist ATL-801 significantly lowers pro-inflammatory cytokine levels	[56]
	IEC	DSS-induced colitis (mouse)	Epithelial-specific A2BR deletion results in a milder form of experimental colitis	[58]
**A3R**	$CD4^{+}$ cells	DSS-colitis model	Global A3R deletion protects from tissue damage, limiting $CD4^{+}$ cells infiltration and preserving colon motility	[59]
	PBMCs	PBMCs from CD patients	Higher levels of A3R are detected in patients' PBMCs when compared to healthy controls	[60]

	Human colonic epithelial cells		A3R activation inhibits NF-kB signaling pathway leading to inhibition of IL-8 and IL-1β pro-inflammatory cytokines	[61]
	Ex vivo inflamed gut tissue	TNBS-induced colitis (rat)	A3R agonist N(6)-(3-iodobenzyl)-adenosine-5-N-methyluronamide limits colitis-induced upregulation of other pro-inflammatory purinergic receptors like P2X1, P2X4, P2X7, P2Y2, P2Y6, as well as A2AR and A2BR	[62]

P2X7R: P2X7 receptor
TNBS: trinitro-benzene-sulfonic-acid
CD: Crohn’s disease
UC: ulcerative colitis
P2Y2R: P2Y2 receptor
P2Y6R: P2Y6 receptor
IEC: intestinal epithelial cells
DSS: dextran sulfate sodium
A1R: A1 receptor
A2AR: A2A receptor
A3R: A3 receptor
A2BR: A2B receptor
DNBS: dinitro-benzene-sulfonic-acid
